# ‘*Valued and listened to*’: the collective experience of patient and public involvement in a national evaluation

**DOI:** 10.1177/17579139221103184

**Published:** 2022-07-14

**Authors:** K Clare, A Ojo, J Teke, M Willis, G Akhtar, B Clegg, C Goddard, C Freeman, KJ Drew, D Radley, C Homer, L Ells

**Affiliations:** Obesity Institute, Leeds Beckett University, School of Health, Leeds Beckett University, Leeds, UK; Re:Mission Patient and Public Involvement Group, Obesity Institute, School of Health, Leeds Beckett University, Leeds, UK; Obesity U, Southport, UK; Re:Mission Patient and Public Involvement Group, Obesity Institute, School of Health, Leeds Beckett University, Leeds, UK; Re:Mission Patient and Public Involvement Group, Obesity Institute, School of Health, Leeds Beckett University, Leeds, UK; Re:Mission Patient and Public Involvement Group, Obesity Institute, School of Health, Leeds Beckett University, Leeds, UK; Re:Mission Patient and Public Involvement Group, Obesity Institute, School of Health, Leeds Beckett University, Leeds, UK; Re:Mission Patient and Public Involvement Group, Obesity Institute, School of Health, Leeds Beckett University, Leeds, UK; Obesity UK, Southport, UK; Re:Mission Patient and Public Involvement Group, Obesity Institute, School of Health, Leeds Beckett University, Leeds, UK; Obesity UK, Southport, UK; Obesity Institute, Leeds Beckett University, School of Health, Leeds Beckett University, Leeds, UK; Obesity Institute, Leeds Beckett University, School of Health, Leeds Beckett University, Leeds, UK; Obesity Institute, Leeds Beckett University, School of Health, Leeds Beckett University, Leeds, UK; Sport and Physical Activity Research Centre, Sheffield Hallam University, Olympic Legacy Park, 2 Old Hall Road, Sheffield S9 3TU, UK; Obesity Institute, Leeds Beckett University, School of Health, Leeds Beckett University, Leeds, UK

This article provides an account of the positive contribution of a patient and public involvement (PPI) team involved in research evaluating the National Health Service (NHS) in England’s low-calorie diet pilot aiming to reduce levels of type-2 diabetes. The article has been co-written by the PPI team and academics from the Re:Mission study. The PPI team members’ voice and experiences are included throughout the article and are reflected using terms ‘our’ and ‘we’.

**Figure:**
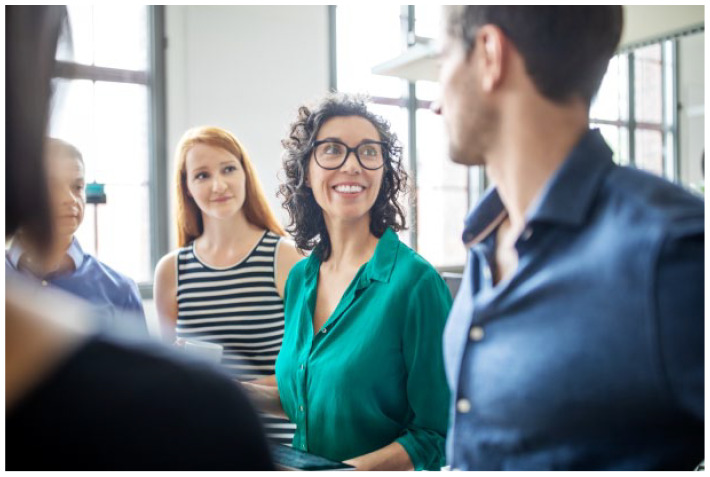


## The Re:Mission Study

Obesity and type-2 diabetes (T2D) are both prevalent non-communicable diseases in the United Kingdom, which can significantly impact people’s health and wellbeing, while leading to significant costs to the NHS and wider economy. Recent systematic review^1^ and clinical trial^2^ evidence shows that for some people living with, or at risk of obesity and T2D, a low-calorie diet achieved by total diet replacement (TDR), can lead to clinically significant weight loss, support remission of T2D and improve quality of life. The NHS long-term plan,^3^ therefore, made a commitment to pilot an NHS low-calorie diet programme delivered through TDR, for people living with excess weight and T2D. In 2020, the National Health Service in England (NHSE) identified 10 initial pilot sites to test the NHS low-calorie diet programme, delivered using one of three different behaviour change support models: one to one, group or digital.

The Re:Mission study (www.remission.study) was commissioned by the National Institute for Health Research to deliver a co-produced, comprehensive qualitative and economic evaluation of the NHS low-calorie diet pilot, that will be integrated with the NHSE quantitative analyses, to provide an enhanced understanding of the long-term cost-effectiveness of the programme and its implementation, equity, transferability and normalisation across broad and diverse populations.^4^ PPI is central to the Re:Mission study, from the preparation of the initial funding proposal, through to the study design, delivery and dissemination.

## What is PPI in research?

PPI is research conducted ‘by’ or ‘with’ members of the public rather than ‘for’, ‘to’ or ‘about’ them.^5^ This can involve contributing to the entire research process from design to the dissemination.^6^ PPI members provide important insights based on their lived experience that researchers may not have considered, but are critical to the end user^7^ and the research process. Without appropriate PPI, resources can be wasted on research that is ultimately not beneficial to end users. Consequently, research funders, such as the National Institute for Health Research, now require PPI as a condition of funding.

## Diversity in PPI and Research

The involvement of members of the public in research is vitally important and should never be seen as a ‘tick box exercise’. Ensuring equality, diversity, inclusion and bringing research to underserved communities is critical, and part of the National Institute for Health Research strategy to achieve ‘the best research for best health’.^8^ As people from diverse ethnic groups are often less likely to take part in clinical research,^9^ it is important to ensure their voice is heard in PPI activity. The Re:Mission study PPI group is an ethnically diverse group, which has been fundamental in the co-development of culturally sensitive research materials, a targeted recruitment process and an inclusive study website. Diversity includes other factors, and the group includes male and female members of different ages and work status from across England.

## Our PPI Role in the Re:Mission Study

The level of engagement of our PPI group can be described as a blend of collaboration (an ongoing partnership with members of the public in the research process) and co-production (a sharing of power and responsibility between researchers and PPI members throughout the study). As PPI members on the Re:Mission study, we were given the opportunity to make comments on anticipated and unanticipated issues that may or may not have been considered by the research team. At the initial design stage, we provided insights into the feasibility of the study, identified potential barriers and facilitators to recruitment, and made suggestions for recruiting ethnically diverse participants and least heard populations. We were able to ensure that the methods selected were appropriate for patients: reviewing and commenting on proposed questionnaires and interview guides, and assisting in the development of study materials. Even the name of the study was suggested and agreed with the involvement of all PPI members.

During the data collection phase, we helped formulate the recruitment strategies and study communications plan, which included visual aids and a short video to inform the public about the study. We have been involved in building the content of the website (www.remission.study), to ensure it meets the diverse needs of the target population and have co-written blogs to communicate research updates. We have all been trained to co-lead participants’ interviews alongside the researchers and will be involved in analysing transcripts to develop the study findings. As PPI members, we have also presented at conferences and seminars, and have been provided with opportunities to co-author journal articles for publication.

## What has involvement in the study meant for us?

Our contribution has been made possible because there has been mutual respect within the PPI group and the research team. Collectively being part of the PPI group has given us a sense of belonging and fulfilment, and a great opportunity to be part of the team. One of the key aspects of working as part of the Re:Mission project has been how we were immediately accepted as valued team members, and how our diverse experiences as patients and stakeholders were recognised as of value. We achieved this despite COVID-19 and all discussions occurring via video conferencing. We quickly forged a positive relationship enabling us to make a tangible contribution that we feel has enhanced the project, particularly where participant-focused. For instance, contributions include the improvement of questionnaire response rates by optimising the flow of the questions and their perceived relevance.

Members have contributed both individually and collectively at all stages of the project. Some points have been immediately accepted by the project team, but on other occasions, we have had to argue the case for changes we recommend, all healthy discussions of course! We have been far from ‘box-tickers’.

Being involved so closely in a fast-paced real-world evaluation has provided us with intellectual stimulation beyond normal working experience or retirement. At all times, we have been thanked for our contributions and have been compensated for our time with prompt payment via gift vouchers, which has been much appreciated!

## What next …

Leeds Beckett University’s Obesity Institute is working in collaboration with the Association for the Study of Obesity and Obesity UK, to continue developing the fantastic contribution PPI makes to improving the impact and reach of research. As such, they are developing a new PPI hub, which will provide an inclusive, supportive and collaborative environment to ensure that PPI is central to all future obesity-related research.

## References

[1] Astbury NM Piernas C Hartmann-Boyce J et al. A systematic review and meta-analysis of the effectiveness of meal replacements for weight loss. Obes Rev 2019;20(4):569–87. 10.1111/obr.12816 PMC6849863 30675990

[2] Lean MEJ Leslie WS Barnes AC et al. Durability of a primary care-led weight-management intervention for remission of type 2 diabetes: 2-year results of the DiRECT open-label, cluster-randomised trial. Lancet Diabetes Endocrinol 2019;7(5):344–55. 10.1016/S2213-8587(19)30068-3 30852132

[3] NHS. The NHS long term plan, 2019. Available online at: https://www.longtermplan.nhs.uk/

[4] Ells L Flint S Martin A et al. A coproduced mixed method evaluation of the NHS England Low-Calorie Diet implementation pilot, 2021. Available online at: https://fundingawards.nihr.ac.uk/award/NIHR132075 10.3310/MPRT2139 40751925

[5] Authority HR . Public involvement, 2022. Available online at: https://www.hra.nhs.uk/planning-and-improving-research/best-practice/public-involvement/ (Last accessed 17 January 2022).

[6] Edelman N Barron D. Evaluation of public involvement in research: time for a major re-think? J Health Serv Res Policy 2016;21(3):209–11. 10.1177/1355819615612510 PMC4904347 26510440

[7] Gordon L Dickinson A Offredy M et al. A research note on the benefit of patient and public involvement in research: the experience of prostate cancer patients regarding information in radiotherapy. Radiography (Lond) 2017;23(2):167–70. 10.1016/j.radi.2017.02.004 28390550

[8] NIHR. Best research for best health: the next chapter, 2021. Available online at: https://www.nihr.ac.uk/documents/best-research-for-best-health-the-next-chapter/27778 (Last accessed 17 January 2022).

[9] Osuafor CN Golubic R Ray S . Ethnic inclusivity and preventative health research in addressing health inequalities and developing evidence base. Eclinicalmedicine 2021;31:100672. 33554078 10.1016/j.eclinm.2020.100672 PMC7846670

